# User-Centered Design of Serious Games for Older Adults Following 3 Years of Experience With Exergames for Seniors: A Study Design

**DOI:** 10.2196/games.6254

**Published:** 2017-01-11

**Authors:** Ellen Brox, Stathis Th Konstantinidis, Gunn Evertsen

**Affiliations:** ^1^ Norut Northern Research Institute Tromsoe Norway; ^2^ School of Health Sciences The University of Nottingham Nottingham United Kingdom

**Keywords:** user studies, usability testing, gestural input, user-centred design, accessibility, consumer health, exergames, participatory design, lessons learned

## Abstract

**Background:**

Seniors need sufficient balance and strength to manage in daily life, and sufficient physical activity is required to achieve and maintain these abilities. This can be a challenge, but fun and motivational exergames can be of help. However, most commercial games are not suited for this age group for several reasons. Many usability studies and user-centered design (UCD) protocols have been developed and applied, but to the best of our knowledge none of them are focusing on seniors’ use of games for physical activity. In GameUp, a European cofunded project, some prototype Kinect exergames to enhance the mobility of seniors were developed in a user-centered approach.

**Objective:**

In this paper we aim to record lessons learned in 3 years of experience with exergames for seniors, considering both the needs of older adults regarding user-centered development of exergames and participation in UCD. We also provide a UCD protocol for exergames tailored to senior needs.

**Methods:**

An initial UCD protocol was formed based on literature of previous research outcomes. Senior users participated in UCD following the initial protocol. The users formed a steady group that met every second week for 3 years to play exergames and participate in the UCD during the 4 phases of the protocol. Several methods were applied in the 4 different phases of the UCD protocol; the most important methods were structured and semistructured interviews, observations, and group discussions.

**Results:**

A total of 16 seniors with an average age above 80 years participated for 3 years in UCD in order to develop the GameUp exergames. As a result of the lessons learned by applying the different methodologies of the UCD protocol, we propose an adjusted UCD protocol providing explanations on how it should be applied for seniors as users. Questionnaires should be turned into semistructured and structured interviews while user consultation sessions should be repeated with the same theme to ensure that the UCD methods produce a valid outcome. By first following the initial and gradually the adjusted UCD protocol, the project resulted in exergame functionalities and interface features for seniors.

**Conclusions:**

The main lessons learned during 3 years of experience with exergames for seniors applying UCD are that devoting time to seniors is a key element of success so that trust can be gained, communication can be established, and users’ opinions can be recorded. All different game elements should be taken into consideration during the design of exergames for seniors even if they seem obvious. Despite the limitations of this study, one might argue that it provides a best practice guide to the development of serious games for physical activity targeting seniors.

## Introduction

### Overview

Physical activity is important at all ages, and seniors particularly need sufficient strength, balance, and flexibility to manage in everyday life. This is particularly true for those ages 65 years and older.

Exergames can be a method to motivate seniors to exercise and hence get more physical activity with sufficient physical exertion [1]. Most commercial games are, however, not suited for this group for several reasons including speed, amount of information, required movements, etc [2-4].

GameUp, a project cofunded by the European Union, Spain, Norway, and Switzerland [5,6] aimed at creating useful and motivational exergames for seniors, was undertaken with a user-centered approach during the design and development process to meet the users’ limitations and requirements.

Despite the fact that many usability studies and user-centered design (UCD) protocols have been developed, to the best of our knowledge none of them have focused on seniors’ use of games for physical activity (ie, exergames). Furthermore, there is limited information regarding the design and functionality of serious games for seniors and more specifically for exergames. An initial UCD protocol was created based on literature and previous research outcomes in order to develop the GameUp project exergames. During the project, we followed that protocol and adjusted it based on 3 years’ experience in order to meet the needs of both the project and the users. In this paper we report the lessons learned for the different phases of the protocol and the adjustments that our initial protocol needed in order to be applicable to older adults and we highlight the most important UCD influences and recommendations on the GameUp exergames.

The remainder of the paper is structured as follows. Initially, we set the scene by pinpointing the need for unique design for seniors, exploring the UCD, and identifying the need for a specific UCD for seniors. In the Methods section we describe all the necessary elements of lessons learned, namely the UCD protocol which served as the base of our exergames implementation, the recruitment criteria for the seniors, the GameUp exergames, and the considered ethical aspects and risks. Next, the Results section describes the lessons learned from a 3-point view: (1) the lessons learned during the different protocol phases, (2) changes and tips for our initial protocol on the different methodologies of the UCD protocol, and (3) the lessons learned from the important changes of our developed exergames. The Results section points out the initial protocol’s weaknesses against the existing literature including the limitations of our approach. Finally, the Conclusions section sums up the lessons learned and emphasizes the key issues on applying UCD with seniors.

### Why a Unique Design for Seniors?

It has been established in several studies that seniors enjoy playing exergames and they believe exergames can assist in maintaining physical activity [7-12]. Despite the fact that balance and rehabilitation of seniors can be maintained and improved through exergames [2,3,7,8,13-16], not many are designed for this age group [11,17,18]. Game designers use their creativity for game ideas, but since designers normally are young, they do not often consider the needs of seniors [19]. The game stories might therefore not be of interest for the senior population.

A trial using Nintendo Wii exergames for seniors indicated that age-related impairments influence the use of video games among frail elderly, so this should also be considered in the design process [20]. We have indeed observed that there are many obstacles for seniors playing commercial exergames, and this has been confirmed in other studies [4,7]. Also, existing games for the young are not developed to meet the physical exercise needs of elderly people. Based on these findings we can conclude that good and safe exergames should be developed particularly for seniors, both regarding movements and narrative.

### What Is User-Centered Design?

The International Organization for Standardization uses the term “human-centered design” and defines it to be an “approach to systems design and development that aims to make interactive systems more usable by focusing on the use of the system and applying human factors/ergonomics and usability knowledge and techniques” [21]. The same standard also states that this term in practice is used synonymously with UCD. According to Karat and Karat [22], “UCD defines iterative processes whose goal is the development of usable systems.” According to Sebe [23], “user-centered design (UCD) is a process (not restricted to interfaces or technologies) in which the needs, wants, and limitations of end users of a product, service, or process are given extensive attention at each stage of the design process. UCD can be characterized as a multistage problem-solving process that not only requires designers to analyze and foresee how users are likely to use a product but also to test the validity of their assumptions with regard to user behavior in real world tests with actual users.”

Since UCD can be considered a multistage process, one will normally use different methods in different stages. Both qualitative and quantitative methods were used by Proffitt and Lange [24] while implementing a UCD. They used focus groups as well as an iterative user-testing process while testing changes to a prototype. One study [25] used a 3-stage qualitative UCD approach in the requirement phase including literature search and focus groups. The actual use of UCD in the industry was studied by Vredenburg et al [26] wherein 13 methods were identified, among them field studies, user requirements analyses, iterative design, usability evaluation, task analyses, focus groups, user interviews, participatory design, and prototypes. Several of these methods have been used in our initial protocol.

### Need for a Specific User-Centered Design for Seniors

Gregor et al [27] further conclude that UCD principles need to be employed for seniors. Seniors are different from the young; “functionality, needs, and wants differ from the young even though they consider themselves as fit, but many often have several physical problems with a general reduction in functionalities” [27]. The authors further refer to the difficulty both of recruiting representatives from this group and of communicating with them.

When users have special needs like the senior users do, the costs of applying UCD increases as the users have more diverse requirements [28]. Zaijcek [29] concluded that it is difficult to arrange traditional focus groups for seniors, which is a common method in UCD. Focus groups should be adapted for older people, and their organization requires considerable interpersonal skills. They conclude that interface design for seniors is more complex than for other groups.

According to Zajicek, “adults as they get older experience a wide range of age-related impairments including loss of vision, hearing, memory and mobility, the combined effects of which contribute to loss of confidence and difficulties in orientation and absorption of information” [29]. With age, eyesight and hearing deteriorate and seniors require more time to think and get an overview [10,30]. Also motoric skills deteriorate with age, and many seniors have health conditions limiting their abilities.

Dickinson et al [31] made a list of guidelines for maximizing the research outcomes of working with older adults. The test case was to learn to use email, but some of the recommendations are also valid for exergame development. One is to put great care into making sure information and instructions are understood; another is that one has to be flexible when it comes to timing during trials. They also point out the difficulties of recruitment and the importance of being able to reschedule and be flexible on timing to maintain participation in a long-term study.

Existing research on Web design for elderly people shows the importance of designing and implementing games and applications uniquely targeting elderly people. We have identified some initiatives and research aimed at providing guidelines for the design and accessibility of websites for elderly people [32-40]. AgeLight [33] points out the importance of player-centered design, meaning that the seniors should be brought in early in the design process. An affordance-based approach to designing a game was followed by Awad et al [41] emphasizing “the type of action the user can perform but also when (response times) and how it can be performed (range of motion).” In this study the authors followed an iterative testing process starting with an early prototype.

Despite the fact that some studies followed a UCD, to the best of our knowledge none of the studies coded and formed a UCD protocol for exergames targeting seniors. In addition, there are few studies that recorded the needs of the older adults either for the creation of exergames or as being part of a UCD.

## Methods

### Recruitment

One of the partners in the project was a senior center, and the participants in the UCD were recruited from its members. In order to ensure that participants would be available throughout the duration of the GameUp project, a regular group was formed that met every second week to play exergames and share a meal. Researchers would participate often but not at all gatherings.

All testing, interviews, etc, were performed at these gatherings. To ensure that the participants were in the target group for the GameUp project and also competent to sign an informed consent, inclusion and exclusion criteria were defined (Textbox 1).

**Eligibility criteria.**

Inclusion criteria:

- Aged 65 years or older
- Risk of falling or history of falling
- Recent illness or surgery
- Impaired strength or balance

Exclusion criteria:

- Cognitive impairment, defined as a Mini Mental State Examination score below 25
- Other disease, illness, or limiting condition that would make inclusion and beneficial use of the system difficult, such as complete blindness, deafness, or severe disabilities

### Definition of a High-Level Protocol for User-Centered Design of Exergames for Seniors

**Figure 1:**
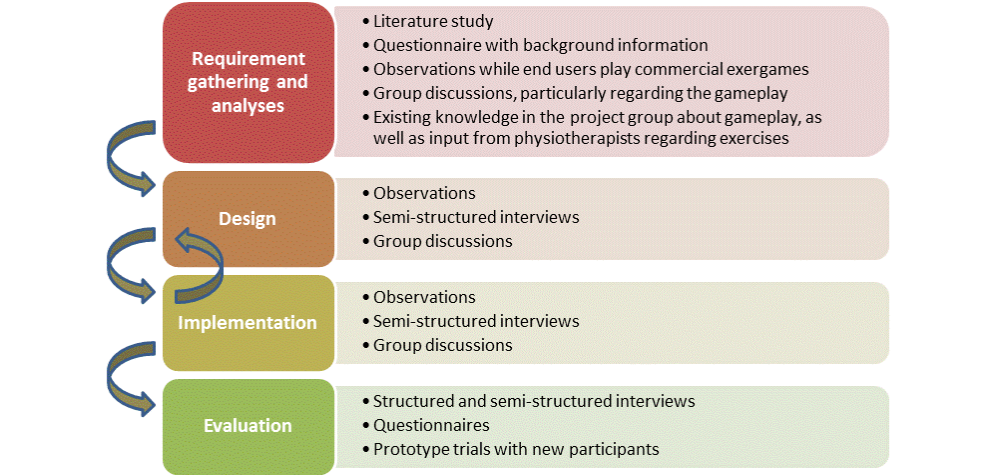
The 4 phases and the methods used in each of the phases.

A high-level protocol for UCD of exergames for seniors was developed by the authors as depicted in Figure 1. This was based on the aforementioned literature and on indications through previous research engaging seniors and exergames on a much smaller scale [10]. This protocol was then tested during the GameUp project on a group of seniors over a 3-year period. A description of the design protocol and how it was used follows.

### Phases of Development

#### Requirement Gathering and Analyses

The main objective of this phase was to gather requirements for an initial design by collecting basic requirements and needs of the end users for the chosen group as well as requirements for the games to be developed by defining useful exercises. The methods for the user involvement in this phase included

- Literature study
- Questionnaire with background information
- Observations while end users play commercial exergames
- Group discussions
- Existing knowledge in the project group

#### Design

In this phase, the initial design of the games become more detailed using an iterative approach. The users should have a real opportunity to influence the outcome of the design by giving feedback. This phase includes the following methods for user involvement:

- Observations
- Semistructured interviews
- Group discussions

#### Implementation

The implementation phase follows an iterative approach based on feedback and user reactions. Detailed descriptions of the design may need to be adjusted merging different parts of the game. During the implementation phase, methods for user involvement are

- Observations
- Semistructured and structured interviews
- Group discussions

#### Evaluation

In the evaluation phase, the emphasis is on the final prototypes. The most common approach is to run pilot tests, but testing the exergame with smaller new user groups can also give valuable input. The methods for user involvement in this phase are

- Structured and semistructured interviews
- Observations
- Questionnaires
- Prototype tests with new participants

### The Games

Microsoft Kinect was chosen for development based on usability studies [42]. The movements included exercises for balance, flexibility, and strength, all important for mastering daily activities.

A total of 7 minigames were developed. The 3 balance games are based on the same concept but with different graphics, and thus they appear as different games. In these games, one is supposed to catch things that fall from above. The falling items (apples, stars, and chickens) are of 2 different colors, and they need to be put in the correct basket. In addition, 4 different minigames for leg strength and flexibility were developed. Since there was a big difference in abilities in the user group, the games have several difficulty levels.

### Ethical Aspects and Risks

Ethical aspects and risks must be identified, including an exit strategy if the participants are enjoying and even getting dependent on the GameUp project results. In our case there were no direct medical interventions, but many exercises are performed standing, and there could be a risk of falling. Some can play alone; for others there must either be a person or chair for support. Some will even play seated or use a walking frame.

A Berg Balance Scale was performed for all the users before the start of the UCD to define the appropriate level of exercises. To avoid any further risk, all the participants were informed about proper use of the system.

The participants have no economic interest or obligations related to the GameUp project, and participants hold the right to exit the project at any time without having to provide a reason for this and without consequences.

Ethical approval for this study was granted by the Data Protection Official for Research in Norway.

## Results

### Study Participants

Approximately 7 to 10 seniors would be present at each gathering, and they were retirees in the age range of 66 to 95 years. In total 16 persons participated.

All participants signed an informed consent and were aware of the fact that they were participating in a research project in which a UCD method would be followed to implement the GameUp exergames. During the 3 years, several members left the group for various reasons, and new ones were recruited. Reasons for discontinuation were varied: 1 moved to a care home far away, a couple got too sick to continue, 1 died, and 1 had a steep cognitive decline and could no longer participate.

The average age was over 80 years but because of the time span and replacements, this was not constant. The first established group consisted of 9 participants, 1 man and 8 women aged 71 to 95 years with an average age of 83 years. Toward the end of the GameUp project at the completion of the UCD protocol, there were 10 participants, 2 men and 8 women in the age range of 66 to 90 years with an average age of 81.7 years.

### Lessons Learned Using User-Centered Design With Seniors

#### Lessons Learned During the User-Centered Design Protocol Phases

##### Requirement Gathering and Analyses

During this phase, a multidisciplinary team should be involved from the beginning. Important inputs for our exergames design were that physiotherapists defined suitable exercises to help the mobility of seniors, developers studied the possibilities of the different tracking movement technologies such as Wii and Kinect, and game designers considered game elements, etc.

##### Design

Videotaping proved to be useful in order to analyze the reactions of the users to different tests. Furthermore, we identified that all the different elements should be taken into consideration during the design of exergames for elderly, including theme, movements, user interface and interaction both with the games and the technology, colors, sounds, playability, etc. As explained above, we developed Kinect-based exercises. During the design phase, we aimed both to learn how the users reacted to this type of interface and which of the physiotherapist-defined movements would be suited for Kinect games. All the different elements were presented progressively to the users. Initially, the first design was presented to the users by presenting graphics on paper and then the interaction with the system was introduced by presenting the physical movements required. Later, design elements like sound and graphics were introduced.

##### Implementation

Detailed descriptions of the design were adjusted during this phase since merging different elements of the design required additional user input. Different tests of the exergames were performed using different methods depending on what was appropriate: (1) test of early prototypes, (2) iterative tests with changes according to outcomes from previous tests, (3) tests of user interface elements (coming together), and (4) test of the playability of the game with focus on game story and game theme.

##### Evaluation

As part of this phase we tested the final prototype with our group. We observed that the group that participated throughout the other 3 phases of the UCD protocol provided feedback during this phase as well, with a few alterations as described in the Study Participants subsection.

#### Lessons Learned Applying the User-Centered Design Protocol Methodologies

**Figure 2:**
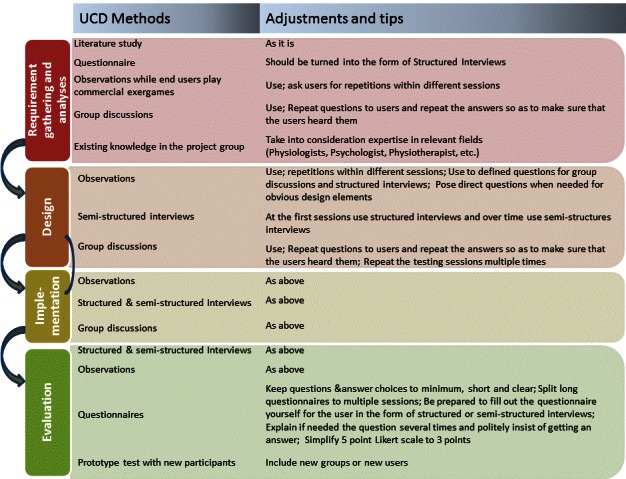
Adjustment to initial protocol for user-centered design (UCD) design having seniors as users.

Our initial UCD protocol was used throughout the project in a UCD methodology although it was slightly adjusted to enable us to reach our goal. The lessons learned could be summed up as a series of tips and adjustments for the creation of serious games for seniors and are summarized in Figure 2.

#### Questionnaires in Interviews

Questionnaires were part of our initial UCD protocol during the “Requirement Gathering and Analyses” and “Evaluation” phases. In order to apply those to our user group, we realized that we had to adjust them by reducing the questions for each session because the users found it hard to concentrate for a long period and they got exhausted very quickly. If there were many questions, we would observe very visible signs of fatigue and loss of concentration.

To this extent, we completed all questionnaires in the form of structured or semistructured interviews since many of the participants had problems both reading and writing and many also had problems understanding some of the questions despite the fact that they met the inclusion and exclusion criteria. Some of the participants also tended to unintentionally skip questions, so the questionnaires have to be short and very clear if used.

When a 5-point Likert scale was used, the facilitators of the session turned the questionnaires into structured interviews as explained above. Furthermore, additional time had to be spent on getting a proper reply. For example, a common answer in this case was “That is fine” in order to avoid a thorough reply. Also the way many responded on color tests gave the impression that they wanted to tell there was nothing wrong with their eyesight instead of telling what they could see the best. Finally a different approach was taken on the questioning than first planned, resulting in minimizing both the number of questions and the number of answering options and spending more time on getting useful answers.

#### Semistructured and Structured Interviews

In the semistructured interviews, we had some open questions and discussions in addition to the structured interviews where we wanted to learn about users’ opinions on specific topics. At the beginning of the application of the UCD protocol, we more or less only got replies to direct questions, which made us think that only structured interviews should be used, but toward the end of the application of the UCD protocol many would give their own suggestions and that allowed us to constructively include semistructured interviews.

#### Observations

Researchers often observed the seniors as they played both commercial games and the GameUp project–developed games. In the observations, we could see how they mastered both the game technology and the movements but also how much they did not perceive. This was particularly the case in many of the commercial games. Based on observations, we also defined questions for group discussions and structured interviews about the project games. For instance, we had the feeling that most could only see exactly what they were doing in the game even if the graphics were very simple and clear. We therefore asked whether they could see information on the top of the screen about the points earned while they were playing. In fact, nobody could see it even though they had been playing the same game several times as well as watching others play. We also observed repeated errors and could adjust the game accordingly.

We used observations to see the users’ reactions to the design elements as well as to detect errors and misunderstandings. Observations also made us change graphics or parameters: the speed of the games or the placement of menu buttons, for example. We also made sure that it was possible to get information before or after gameplay instead of during the game.

#### Group Discussions

**Figure 3:**
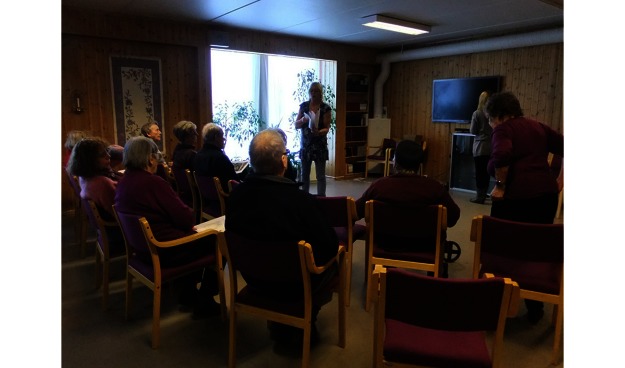
Seated in front of the screen ready to play.

The setting of the group discussion was as follows: the entire group would sit in a semicircle in front of the screen with the person playing in front, and they would take turns playing (Figure 3). In between playing or before and after we would initiate small group discussions while all were seated by triggering a discussion through questions about the game, particularly if we had introduced something new. We found the setting appropriate since the users didn’t have to change places between observing or playing the game and discussing. Issues we brought up during the group discussions included hearing issues of the users. Hearing is a problem for many elderly people; we experienced that we often had to repeat questions and frequently we had to repeat the responses so that all could hear what had been said.

Another important lesson learned is that replies would often come gradually playing the same game version in several sessions. Repetitions were also important to get the response from as many as possible, since there were always some who were not present at specific gatherings.

**Figure 4:**
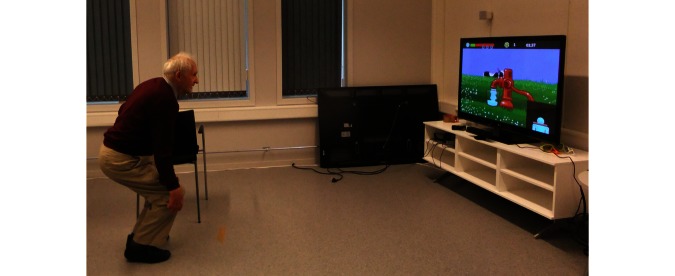
Bending knees to get water.

As an example of the outcome of group discussion, one theme that was successfully discussed was the perceived contents or game story. In one minigame the players perform knee bends, which results in water coming from a pump and into a bucket (Figure 4). Occasionally a cow would pass by and bellow, so many players wanted the water to run into a trough for the cows instead of a bucket. Another example is a flexibility game where the players use a scythe. Most of the players had used scythes in their youth and were a bit frustrated that it was cutting in the wrong direction. In the game, the corn was cut when the scythe was on the way back, but in reality you have to cut on the first move and then swing the scythe back.

**Figure 5:**
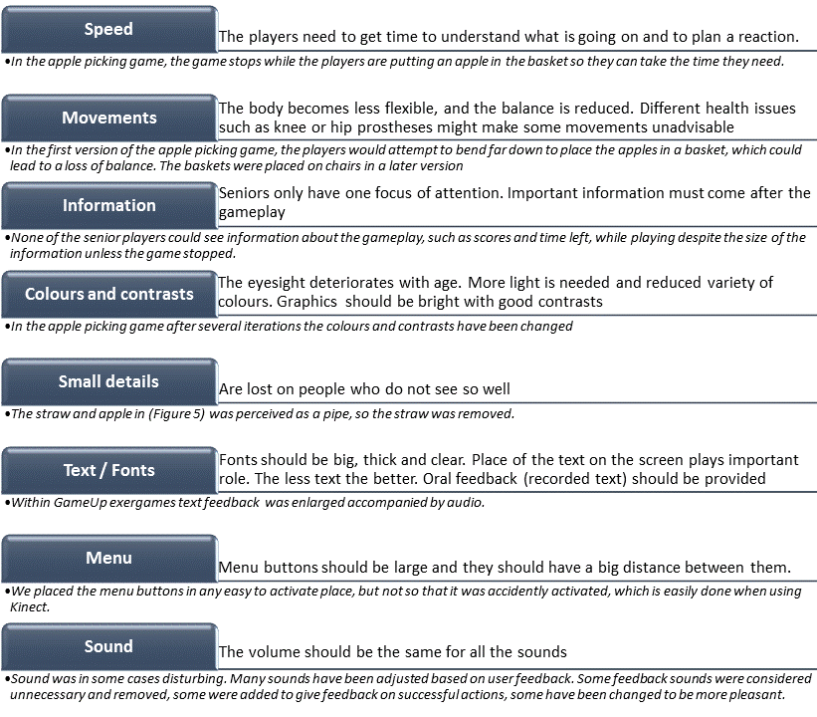
Most important exergames elements and functionality as resulted from 3 years of experience with exergames for seniors.

Many changes occurred to the developed exergames through the 3 years of the GameUp project. We briefly describe the ones that can be generalized to other games in Figure 5.

#### Lessons Learned From Exergames Targeting Seniors—Important Changes

Among the most important elements of the games were speed, both of some movements and of the game progress. In our exergames, we needed to reduce the speed of both and in addition adapt the game to the physical ability of the seniors, so we changed movements that were difficult for many elderly people to perform.

**Figure 6:**
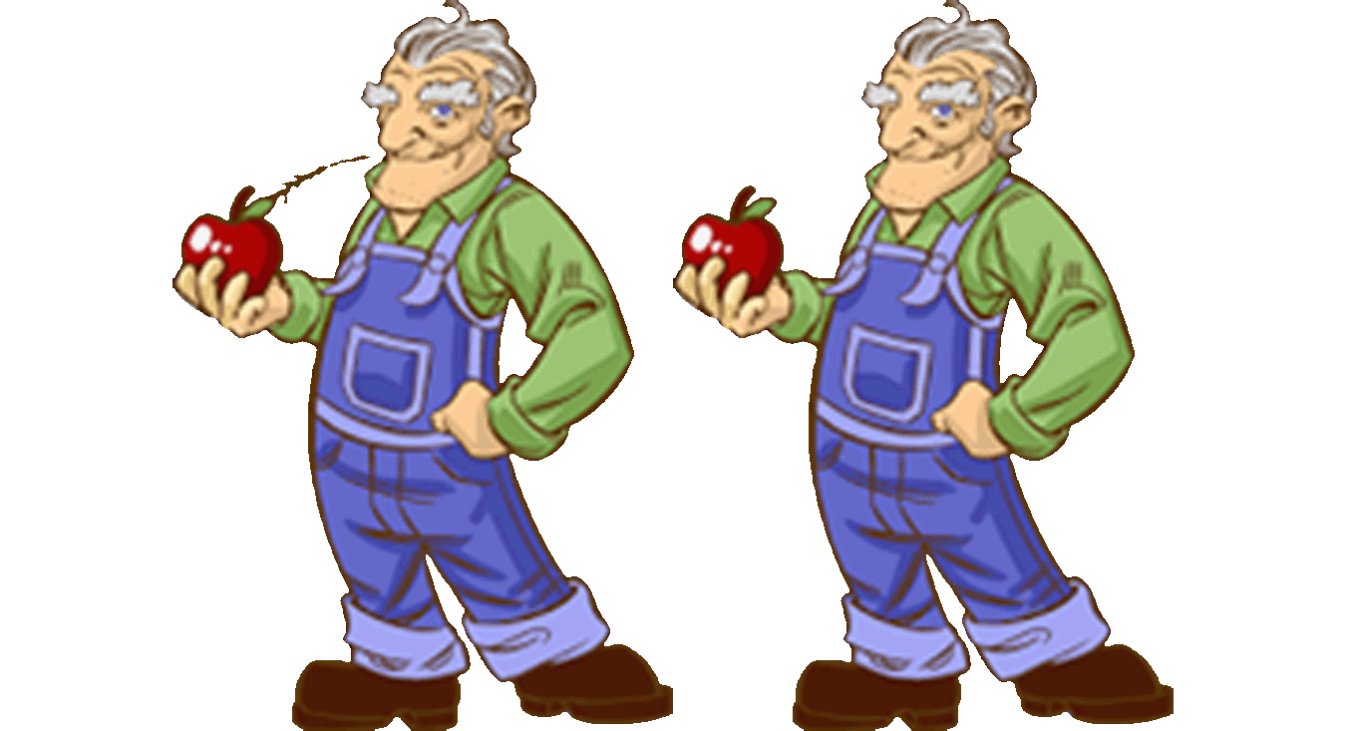
The first and a later version of an “Old farmer” character. With the straw, the players thought he was smoking a pipe.

Eyesight deteriorates with age. We need more light the older we get, and we also see fewer colors. This means that graphics need to be clear and bright, and users’ feedback helped us change graphics. Also small details were lost. For instance, the straw and apple in Figure 6 was perceived as a pipe, so the straw was removed. This also means that fonts must be big and clear, and there should be as little text as possible, preferably accompanied by oral feedback. Oral information is important, but sound can also be very disturbing, and many sounds have been adjusted based on user feedback.

Several menu buttons were enlarged and the distance between them increased. A menu color test was performed to find good color combinations between button, background, and text.

## Discussion

### Prinicipal Findings

Lately research has been focusing on older adults over 65 years, including how to keep them active. Exergames seems to be a promising tool to enable elderly people to be active, but the creation of the games can be difficult since no specific guidelines exist. As participatory design is central to the creation of serious games, in this paper we propose an adjusted UCD protocol tailored to senior users and provide and discuss the lessons learned of applying this protocol over almost a 3-year period.

Taking into consideration the limitation and the cross-validation of findings through published literature, the novelty of this paper lies in the proposal of a UCD protocol and the use of its methodologies tailored to seniors’ needs, lessons learned during the creation of an exergame applying a UCD approach which then can be generalized and applied in serious games targeting seniors, and lessons learned from the application of such a protocol having seniors as users. This may act as a guide for future studies and projects.

Most (exer)game developers are young, and it is difficult for the young to realize the limitations of age. This applies to graphics as well as speed and movements. It is therefore highly advisable to apply a user-centered approach when designing for seniors. It is difficult to recruit the very old, but the authors believe that the users should be as close as possible to the intended user group both in age and abilities. Senior users are often defined as being 55 years and older, but there is often a huge difference between a 55-year-old person and a 95-year-old person both in cognitive and physical abilities. In the 3-year period using user-centered design, the average age of our users was over 80 years.

One big challenge was to ensure that the participants understood the questions we posed and gave an accurate reply. For instance, the nuances were lost on a 5-point Likert scale; we propose using only 3 points when this will still give valid results (eg, “I agree,” “I do not agree,” and “I do not know”).

Observations are very important and should be emphasized throughout the protocol, including hints for the observers about what to look for. This should include errors made during the test but also signs that the participant does not understand or cannot see details, whether there are games or parts of games they do not want to perform, etc. Filming the different tests and sessions can be of great help for later analyses.

Since group discussions can be a challenge and participation was irregular, we developed small questionnaires when we wanted to make sure that we got the opinion of as many as possible and repeated the sessions in several gatherings to get responses from most participants, sometimes even asking selected persons to arrive before the gatherings started. Further, in the group discussions we took the time to direct questions at each and every one, coming up closer to the persons to make sure that they could hear what was being said.

The very old get exhausted easily, so there should not be too many questions and certainly not too many that are almost the same. We particularly experienced this when going through color tests. We had 4 sheets, each with 5 menu buttons, and we wanted to know which ones they could see the best. Toward the end most were exhausted and did not want to continue. Tests like this should only have small samples and could be performed in more than one session.

Working with seniors requires trust, which takes time to build. The group size should not be too big for several reasons: it is difficult to recruit in this age group, the participants need to gain trust inside the group, and group discussions among the old are challenging.

Since many in this user group are more or less computer illiterate, it is also important that they understand that crashes and errors are not their fault but the developers’.

According to Faulkner [43], the number of users in a usability study probably influences the problem discovery level a study will achieve. He demonstrated that 10 users are able to identify a minimum of 82% of the problems with an average problem finding of 94.686% while this percentage changes to a minimum of 90% and an average of 97.050% problems identified by 15 users. Nielsen [44] advises that 5 participants is optimal for problem discovering, while Spool and Schroeder [45] support that problem-discovering relates to the complexity of tasks and 5 participants can only identify 35% of the problems in an interface. As Macefield [46] demonstrated there is no “one size fits all” solution; however, 5 to 10 participants is a sensible baseline range for problem discovery while comparative studies aiming at statistically significant results should have 10 to 12 participants. Furthermore, the length of the study should be taken into consideration. According to Baek et al [47], users participate in a design at the full inclusion level and the emancipatory level by cooperating with the researchers and developers or even carry out the design themselves over a long period of time. Taking into consideration the average age of the participants and the place of the study, the number of users can be considered satisfactory and beneficial for this study.

### Limitations

There are some limitations in our study. The 3 most important are

- The adjusted protocol was modified during a long period with 1 user group. It should be tried on new groups to be confirmed and further adjusted.
- Gaining trust from the participants and also within the group of participants can lead to biased results from participants wanting to please the researchers or developers.
- A small steady group could after a while feel that the game is partly their creation, particularly when they see changes based on their feedback. This can make the users less critical.

### Comparison With Prior Work

The players often bring their own context into game play and this should be reflected in exergames for elderly people [19]. The senior population, for instance, often has other preferences than the young when it comes to music and activities that would make a game enjoyable. Also, activities from the past that can trigger fond memories could make a good background for game stories, but as our example with the scythe shows, elderly people need to be involved to get the stories and activities correct.

When approaching the very old, one needs to be patient and spend the time that is required to gain trust, but tests and interviews will take longer, and less can be done at a time. Our experiences forming, evaluating, and adjusting the proposed protocol echo the findings of Redish and Chisnell [48] that recruiting and working with older adults requires special communication.

Questionnaires are found difficult to read and fill in. According to our experience, when given a paper questionnaire many will ask for help to read, explain, and write the replies. If the seniors are left alone filling them in, there are often many blanks and several replies that indicate that they have not understood the questions. Hayes et al [49] also confirm that seniors often have problems filling in quite simple questionnaires on their own, either feeling unable to do so or missing out on questions or information given. Questionnaires should therefore be filled in by a helper or in structured interviews. This could lead to biased results since they will not be anonymous, but we think that this is a smaller problem than not understanding the questions and one that can be taken into account when analyzing the results.

Iacono and Marti [50] point out how important it is to create an empathic and trusted relationship between participants and designers in participatory design with seniors and state that the role of the facilitator is crucial. Being in line both with our experience and the view of Iacono and Marti is that knowledge is not acquired once and for all by older adults, since they often forget recent events.

### Conclusions

Involving seniors in the entire process can lead to many big and small changes that are essential to make good, safe, and fun games that the seniors will want to play and thus be motivated to exercise more. In this respect, involving senior end users when designing exergames for this group is essential. However, the UCD must be adjusted to suit this user group.

It is important to plan the sessions so as not to exhaust or confuse the participants, and each session should be short and should not cover too many aspects at once. It is also a good idea to leave time to perform the same tests or questions/interviews in several sessions in case many participants are absent at the first one. Also, questions should be formed with care and questionnaires should be short and either filled in using structured interviews or with a helper at hand.

Devoting time to seniors is a key element of the success of a UCD so that trust can be gained, communication can be established, and users’ opinions can be registered. Thus, with some adjustments regarding time and tasks to perform, our initial protocol was useful and gave valuable results.

In the development of exergames for seniors applying UCD, all the different game elements should be taken into consideration during the design of exergames for seniors even if they seem to be obvious. Those elements include theme, movements, the user interface and interaction both with the games and the technology, colors, sounds, playability, etc.

Despite the fact that it might be considered a limitation, another reason for the useful feedback might be that a stable group of senior users participated in our study. They felt safe both with each other and with the researchers. The researchers gained their trust by spending much time with them playing, chatting, and sharing meals and learning the names of all participants.

It is clear from this research that best practices have been formed for UCD of serious games for older adults that look promising for researchers and developers and for facing societal challenges like active and healthy aging as well.

## Abbreviations

UCD: user-centered design
